# Meat transfer patterns reflect the multi-level social system of Guinea baboons

**DOI:** 10.1016/j.isci.2025.113619

**Published:** 2025-09-20

**Authors:** William J. O’Hearn, Christof Neumann, Roger Mundry, Federica Dal Pesco, Julia Fischer

**Affiliations:** 1 Department of Primate Cognition, Georg-August-Universität Göttingen, Johann-Friedrich Blumenbach Institute, Göttingen, Germany; 2 Cognitive Ethology Laboratory, German Primate Center, Göttingen, Germany; 3 Leibniz ScienceCampus Primate Cognition, Göttingen, Germany; 4 Center for Research in Animal Behaviour, University of Exeter, Exeter, UK

**Keywords:** Wildlife behavior, Biological sciences, Zoology, Evolutionary biology

## Abstract

Multi-level societies, characterized by stable subunits nested within higher-order social levels, occur in humans and several other taxa. In hunter-gatherers, resource sharing, particularly meat, is considered a key driver of multi-level social organization. Despite its importance in humans, patterns of resource transmission in non-human multi-level societies remain largely unexplored. Using Guinea baboons, *Papio papio*, as a model, we examined how meat, a high-quality, shareable resource, is transmitted through their multi-level society. We combined records of 109 meat-eating events with nine years of behavioral and demographic data to test how relationship strength and stratified social levels affect meat sharing. Meat transfers were more likely to occur along stronger social relationships, and tolerant transfer types were most common at the society’s base, decreasing with each higher social level. This pattern resembles that of some hunter-gatherer societies, suggesting convergent outcomes of multi-level social organization on the transmission of high-quality shareable resources.

## Highlights

• Meat transmission pattern is explored in the multi-level society of Guinea baboons
• Tolerant meat transfers are most common at the base of the society
• Relationship strength facilitates the transmission of meat
• Meat transmission follows patterns similar to multi-level hunter-gatherer societies

## Graphical abstract

**Figure:**
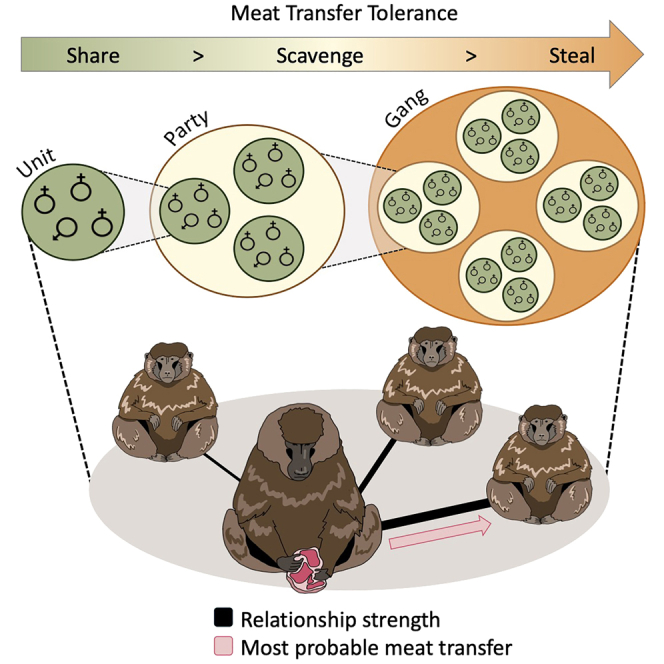

## Introduction

In a multi-level society, individuals form stable social subunits that are themselves embedded within progressively larger, hierarchically structured social levels.^1^ Among species that live in multi-level societies, human hunter-gatherers are unique for their reliance on the widespread sharing of hunted meat, which not only defines their essential ecological niche,^2^^,^^3^^,^^4^ but is also thought to have prompted their shift to multi-level organization.^5^^,^^6^^,^^7^^,^^8^^,^^9^ The variable success of hunting, combined with the dietary value and high yield of hunted food, is thought to have encouraged early humans to develop sharing networks between households to ensure a regular supply of meat.^7^^,^^8^ The size and composition of intermediate social levels (clusters) are often seen as a balance between the number of households needed for regular reciprocal food sharing and the tendency to fission when numbers grow too large and competition too frequent.^10^ The result is a society with multiple nested levels throughout which food is shared - households within bands, bands within a camp.^3^^,^^5^

The means and paths by which meat is shared between individuals throughout the multiple nested levels of hunter-gatherer societies follow surprisingly consistent patterns,^6^^,^^7^^,^^11^ even between geographically distant communities.^12^ Meat is transferred along existing social relationships both within and between households. Similarly, meat sharing is most prevalent at the base of societies, decreasing in frequency as meat is shared outward across successive social levels.^11^^,^^12^^,^^13^^,^^14^^,^^15^ The transmission of high-quality resources, such as meat, is well studied in humans. Yet it is largely unstudied in other multilevel societies,^1^^,^^6^^,^^16^ which raises the question of whether the pattern of meat sharing recorded in humans is unique or whether a similar pattern of resource transmission may emerge in other multi-level societies.

To address this question, we examined the pattern by which meat is transmitted through the multi-level society of Guinea baboons, a non-human primate that catches prey and tolerantly transfers meat.^16^ Guinea baboons live in a society consisting of nested social levels very similar to that of some hunter-gatherer communities. The lowest societal subunit (baboon: “unit,” human: “household”) comprises one male, one or more associated females, and their offspring.^12^^,^^13^^,^^17^ The second societal level (baboon: “party,” human: “cluster”) comprises three to four subunits linked by enduring male-male bonds underpinned by kinship.^17^ The third level (baboon: “gang,” human: “camp”) is a diffuse collection of individuals more weakly connected by social ties or kinship than the two lower levels.^9^^,^^12^^,^^18^^,^^19^^,^^20^ The evolutionary origin of the Guinea baboon’s multi-level social organization remains a subject of ongoing research. Of particular interest are the circumstances that promoted the transition from ancestral female-philopatric uni-level societies to the male-philopatric multi-level social organization of Guinea and hamadryas baboons.^21^ According to Jolly’s “philopatry at the frontier” hypothesis, this shift occurred during the northward range expansion of the species.^22^^,^^23^

Like other members of the genus, Guinea baboons are highly opportunistic, eclectic omnivores.^24^ Their diet consists of a wide range of woody and herbaceous plants, fruits, invertebrates, and occasionally birds and small mammals.^16^^,^^24^^,^^25^ Guinea baboons opportunistically chase and catch prey,^16^^,^^26^ primarily young bushbuck (*Tragelaphus scriptus*), representing a relatively large prey item for the baboons (young bushbuck: 10–14 kg, adult Guinea baboon: 10–26 kg). While Guinea baboons do not actively share meat, they do exhibit a tolerant form of meat transfer that cannot be explained by harassment avoidance.^27^ Once caught, prey is often fed on by multiple baboons one at a time, with the carcass being transferred between many individuals in sequence.^16^ Transfers between individuals can differ in their degree of tolerance exhibited between the possessor and the recipient (see Table 1). The most tolerant transfer type is “passive sharing,” whereby the recipient approaches the owner and removes meat from the ground nearby or the carcass itself. There are two neutral transfer types: “scavenging” is when recipients take pieces of meat left behind when the possessor moved the carcass, and “succeeding” occurs when a recipient moves into the feeding position vacated by the owner. “Stealing” transfers are agonistic, where the recipient forcefully takes the possession of the carcass despite resistance by the owner.^16^ It is worth noting that Guinea baboons transfer meat more often and more tolerantly than other baboon species, including the other multi-level hamadryas baboon.^16^^,^^26^^,^^28^^,^^29^ This difference is likely due to their generally tolerant disposition.

**Table 1:** Definitions of meat eating transfer types

Transfer type	Description
Passive share	a recipient took meat from the carcass itself or the ground just around the carcass (<2 m) while the owner was still feeding and in the absence of aggression and submission by either individual
Succeed	recipient moved into the feeding position recently vacated by the owner (formerly ‘supplant’^16^)
Scavenge	Recipient a acquired pieces of meat left behind (>2 m) when the possessor moved the carcass
Steal	the recipient forcefully took the possession of the carcass despite resistance by the owner

Our study aims to examine how meat moves through the Guinea baboon’s multi-level society and to test whether societal levels and social relationships structure the transfer of meat. Specifically, we investigate the extent to which different transfer types, ordered by their degree of tolerance, map onto the levels of Guinea baboon society. We also test whether an individual’s probability of getting meat is predicted by the strength of their relationship with the meat’s possessor. In this way, one analysis describes the behavior by which meat will be transferred, and the second analysis describes the path by which the meat will move. In combination with descriptive statistics from an extensive collection of meat-eating events, our analyses describe the patterns of meat transmission in a non-human multi-level society. Given the prevalence of compartmentalized behaviors in other multi-level systems, we expect to find some degree of stratification in transfer type by social level.^30^ Similarly, we predict that meat transfers in Guinea baboons will flow along strong social relationships due to their generally more cooperative nature.^31^^,^^32^^,^^33^^,^^34^ By comparing the meat sharing patterns of Guinea baboons with those found in humans, we aim to determine whether certain features of meat sharing are generalizable across multi-level societies.

## Results

To map the transmission of meat through the multi-level society of Guinea baboons,^19^^,^^35^ we recorded transfers during meat-eating events, analyzed how the tolerance of transfer types was affected by societal level (units within parties within gangs), and examined how social preference from a meat possessor affected an individual’s likelihood of receiving meat. Over nine years, we collected data on meat transmission in wild Guinea baboons ranging near the field site CRP Simenti in the Niokolo Koba National Park, Senegal.

**Figure 1:**
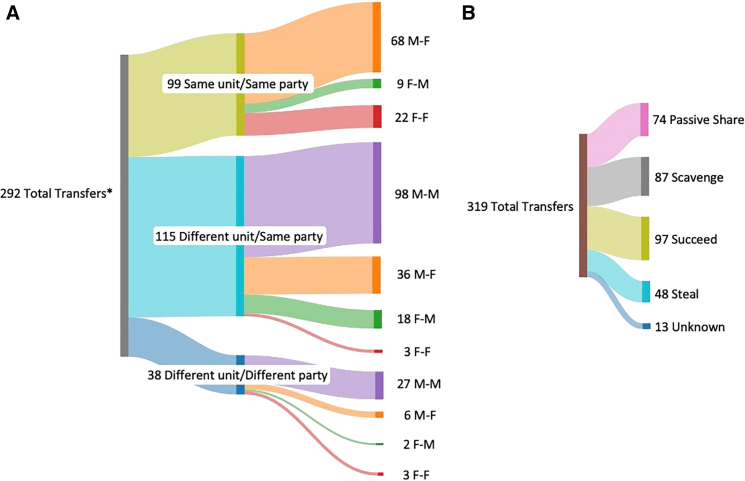
Guinea baboon meat transfers by possessor/recipient sex and transfer type Sankey diagrams show meat transfers split by (A) shared social level and sex of the possessor and recipient and (B) transfer type. ∗ refers to all transfers for which we knew the unit membership and sex of both possessor and recipient.

We recorded 109 meat-eating events between April 2014 and June 2023, in which 103 individuals from 13 parties participated as possessors or recipients. Within the 109 meat-eating events, we recorded 320 meat transfers, with a median value of two transfers per event (range: 1–17). In 22 events, the individual who caught the prey consumed the entire prey and did not transfer any meat. In all of these 22 cases, the prey were small birds (Table S1). Of the transfers between adults for which we knew the unit membership of both possessor and recipient (*N* = 292), transfers between males were most frequently observed, representing 42.8% (125/292) of total transfers and occurring most often between males of the same party (98/125; Figure 1A). Male-to-female transfers were next most common, representing 37.7% (110/292) of transfers, and occurred most frequently at the unit level between males and their associated females (68/110; Figure 1A).

### Tolerant meat transfers were most common at the base of the society

**Figure 2:**
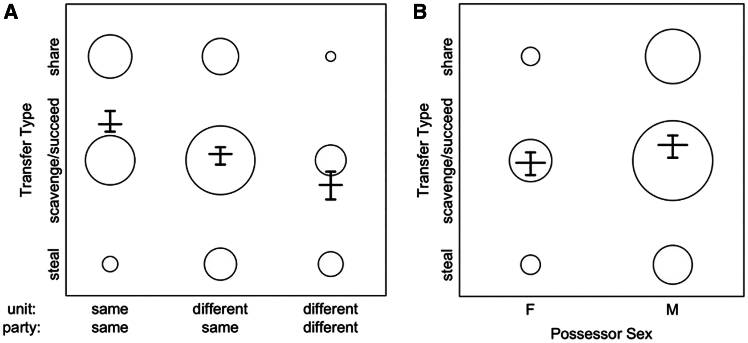
Tolerant transfer types were more common closer to the society’s base Transfer types of decreasing tolerance (share > scavenge/succeed > steal) and how their frequency varied depending on (A) the shared social levels of meat possessor and recipient and (B) possessor sex. The area of the bubbles shows the relative frequency of transfer types [ranges: (A) 2–101, (B) 7–135]. Vertical lines with error bars depict the fitted model and its confidence limits (with all other predictors being at their average).

To test how the tolerance of meat transfers was related to the level of the society at which they occurred, we fitted an ordinal mixed model^36^ with three transfer types ordered from most to least tolerant (share > scavenge/succeed > steal; transfer type frequency is shown in Figure 1B). The model contained data from 62 meat-eating events, involving 283 transfers between 65 possessors and 79 recipients across 152 dyads. The model performed significantly better than the null model (χ^2^ = 33.624, df = 5, *p* < 0.001) and showed that more tolerant transfer types occurred more frequently at the unit level (same unit/same party; Figure 2A) compared to the party level (different unit/same party: Estimate = 2.345, SE = 0.539, *P* = < 0.001; Table S2). Transfers were similarly more tolerant at the party level compared to the gang level (different unit/different party: Estimate = 1.224, SE = 0.438, *p* = 0.005). In other words, more tolerant types of meat transfers (share > scavenge/succeed > steal) were most common at the base level of society and decreased in frequency at higher social levels (Figure 2A).

We found no significant effect of the interaction of possessor and recipient sex (Estimate = 0.738, SE = 0.657, *p* = 0.260; Figure 2B; Table S2), indicating both sexes were equally likely to transfer and receive meat tolerantly. However, in a reduced model lacking the interaction of possessor and recipient sex, males were slightly more likely to transfer more tolerantly than females (Estimate = 0.711, SE = 0.365, *p* = 0.051; Table S3), and recipient sex was not significantly related to the tolerance of the transfer type (Estimate = −0.428, SE = 0.377, *p* = 0.246; Table S5).

### Social relationship strength predicts the probability of receiving meat

To test whether close social associates of meat possessors were more likely to receive meat when in the audience of a meat-eating event, we built a custom Bayesian model fitted in Stan. Using behavioral data (number of approaches, grooming duration, and contact sitting duration) we first estimated the “dyadic affinity” for each pair of individuals - a measure of the preference of two individuals to interact with one another while controlling for individual gregariousness.^37^ Using a subset of transfers, we modeled the probability of an individual obtaining the meat given their affinity with its current possessor. The subset excluded transfers for which we could not calculate the dyad’s affinity because dyad members resided in different parties and all “steal” type transfers, as these did not reflect the possessor’s motivation to share. The final model used 232 meat transfers and individuals from eight parties, including 59 possessors, 68 recipients, and 83 audience members. Audience members were defined as identified individuals in the area surrounding a meat possessor (∼10-meter proximity).

From the two control sub-models of our custom Bayesian model, we found a positive but uncertain relationship between individual gregariousness and audience size (posterior median = 0.13; Table S5; Figure S2) and a positive relationship between dyadic affinity and audience composition (posterior median = 0.14; Table S5). Thus, individuals who were generally more gregarious in focal observations also had slightly larger audiences during meat-eating events, and individuals with a higher affinity to the meat possessor were more likely to be in the audience than the rest of the party.

**Figure 3:**
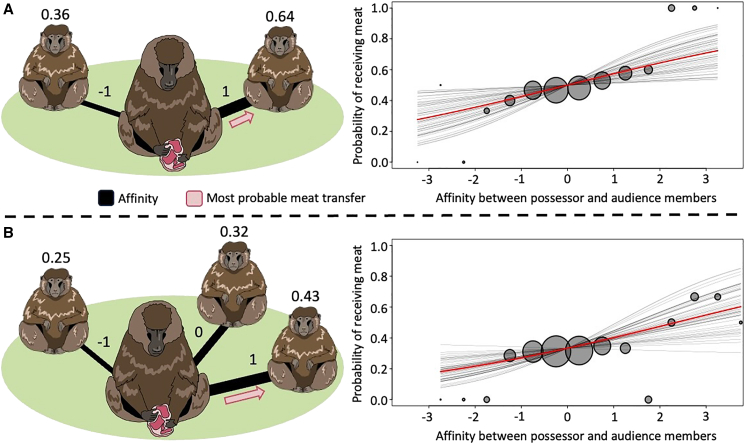
Audience members with a stronger affinity to the possessor were more likely to receive meat The bubbles show the observed proportions of cases when an audience member of a given affinity with the possessor received the meat. Bubble size is proportional to the number of observations in a given bin of affinity (bin width = 0.5). (A) and (B) reflect the two most common audience sizes of 2 and 3 with 64 and 39 cases, respectively. The red lines show the predicted relationship (posterior median) between affinity and the probability of receiving meat, and black lines show predictions from 50 posterior draws. The probability of each audience member participating in a meat-eating event is affected by their affinity to the meat possessor and the size of the audience. Audience members with stronger affinities are more likely to receive meat, but a larger audience size dilutes the effect. The probability of each audience member obtaining the meat was calculated using the SoftMax function, which creates a set of probabilities that sum to one based on the number of audience members and their dyadic affinity values with the meat possessor. (A) SoftMax (0.30 × [−1.0, 1.0]) = [0.36, 0.64]; (B) SoftMax (0.30 × [−1.0, 0, 1.0]) = [0.25, 0.32, 0.43]. Drawings by Maggie Slowinska. See also Table S5 and Figure S2.

In our primary sub model, we found an overall positive relationship between affinity and an individual’s probability to receive meat (posterior median = 0.28, 89% credible interval = 0.07–0.56; Figure S2). Thus, having a stronger dyadic relationship with the meat possessor increased the likelihood that an audience member would receive meat. To illustrate this result and its effect size, we present two scenarios. Suppose there is a meat-eating audience of two individuals, reflecting the median audience size (range: 1–6), with affinities to the possessor slightly above and below average (−1.0 and 1.0). All else being equal, the estimated probabilities of each individual receiving meat with a slope estimate of 0.30 would be 0.36 and 0.64, respectively (Figure 3A). With an audience of three individuals possessing the affinities −1.0, 0, and 1.0, the resulting probabilities of getting meat would be 0.25, 0.32, and 0.43 (Figure 3B). These examples illustrate how the probability of each audience member receiving meat increased with greater affinity to the possessor and how that probability was modulated by the audience’s size and relative affinities. If there were multiple individuals in the audience with high affinity, if differences in affinity were minor, or if the audience was large, the affinity effect was diluted. In such circumstances, all audience members could have similar chances of getting meat. Meat transfers in Guinea baboons are thus modulated, but not determined, by social relationship quality.

## Discussion

In Guinea baboons, prey was most frequently caught, and meat was most commonly transferred by males, specifically to males within their parties and females within their units. Transfers of meat were more likely to occur along stronger social relationships, and the type of transfer became less tolerant at successively higher levels of the multi-level society. We did not observe any active “possessor-initiated” transfers of meat, such as those recorded in chimpanzee (*Pan troglodytes*) begging circles,^38^^,^^39^ vampire bat (*Desmodus rotundus*) blood sharing,^10^ or human meat sharing.^13^^,^^40^ However, we did observe tolerant transfers of meat between parties, making this one of the few documented cases of food sharing across social units in a non-human species, along with inter-community meat sharing in bonobos^41^ and resource exchanges in polydomous ant systems.^42^

Meat appears to be transmitted throughout the Guinea baboon society passively via tolerance along gradients of familiarity within social levels between dyads. The mode of meat transmission is perhaps best exemplified by the “queuing behavior” we frequently observed during meat-eating events in which one individual possessed a carcass and ate from it while audience members sat nearby. These animals often greeted or groomed one another until the owner was satiated and left the carcass. At this point, one individual, usually the one seated closest, would approach the carcass and begin eating. This process could repeat until all individuals had fed or the group moved off. Individuals in the queue with higher relationship strength likely experienced greater spatial tolerance from the meat possessor,^35^ allowing them to sit closer than others and affording greater opportunity to take pieces of meat from the carcass (“passive share”), from the ground (“scavenge”), or to take the possession of the carcass when its possessor left (“succeed”). In this way, the effects of greater tolerance within units and greater transfer probability for close associates can be understood as passive transmission, whereby familiarity begets tolerance, and tolerance begets meat.

We also found that in Guinea baboons, the meat transfer behaviors were stratified by social level along a gradient of tolerance. This compartmentalization of tolerant behaviors is notable for its resemblance to recent findings that stratified forms of helping behavior map discretely onto the social levels of multi-level societies in humans, birds, and cetaceans.^12^^,^^43^^,^^44^ For example, superb fairy wrens respond to distress calls with differing degrees of cooperative assistance, from high-to low-risk, depending on whether calling individuals belong to the same breeding group, the same community, or a different community.^8^ From an evolutionary standpoint, it is of particular interest whether stratified cooperative relationships are a cause or a consequence of multi-level social organization. In the case of Guinea baboons, if one assumes that the switch from uni-level to multi-level social organization followed from the formation of long-term bonds between males and females, resulting in the establishment of “units,” then cooperative relationships would constitute a consequence. However, there are likely co-evolutionary processes at play, with increased relatedness among males promoting social tolerance and cooperation with parties.^17^ An intriguing question for future research is whether multi-level societies create specific in-group-out-group dynamics within their respective levels that differ from those found in clustered uni-level societies.

The pattern of meat transfers in Guinea baboons bears some resemblance to patterns of meat sharing recorded in human hunter-gatherer societies, such as the Agt, Mbendjle, and Hadza.^12^^,^^13^ In those communities, meat transfers have been shown to flow from successful male hunters to females and kin within their households, and then to transfer along a few strong relationships to individuals in other households, before spreading diffusely to the camp level.^12^^,^^20^^,^^45^^,^^46^^,^^47^ The emphasis of male hunters on provisioning their own households with meat rather than sharing the majority of a kill with other households as a “common good” varies between modern hunter-gatherer communities.^13^^,^^48^^,^^49^ In Guinea baboons, we observe the same pattern of transmission with an emphasis on transfers to closely bonded males in other units rather than provisioning unit females.

Despite stark differences in the mechanisms by which their societies facilitate meat transfers and the impetus for their developing multi-level organization, both societies appear to share the pattern in which they transmit shareable foods. Meat brought into the multi-level society by one individual appears to diffuse along gradients of familiarity first within, then between, units and households, moving from base to top, whether through active or passive sharing. Like behaviors stratified by societal level, the similarity of meat transmission patterns in humans and Guinea baboons may reflect the shared properties of hierarchically branching networks.^18^^,^^50^ Common features of social organization, such as scaling ratios between sub-unit sizes in successive social levels,^50^^,^^51^ few key connections between sub-units,^52^^,^^53^ and the decrease of familiarity across levels of an egocentric network,^54^ may result in shared patterns of resource transmission across multi-level societies. In conclusion, our study of meat transmission in Guinea baboons provides insights into how multi-level networks can redistribute high-quality resources, irrespective of evolutionary origins.^6^^,^^32^^,^^33^

### Limitations of the study

This study uses indirect methods to test how meat moves through the multi-level society of Guinea baboons. The historical meat-eating data in this study spans select periods and parties where behavioral data was absent or too sparse to estimate the full social networks of the parties. Consequently, we had to focus on smaller social features that might affect transmission such as transfer type, discrete social levels, and social preference. Future analysis with more accumulated meat-eating events, accompanied by more complete behavioral data across time and parties, would enable the use of tools such as network-based diffusion analysis to determine if the transmission of meat through the multi-level society is predicted by specific, measurable features of Guinea baboon social networks.

## Resource availability

### Lead contact

Requests for further information and resources should be directed to William J O’Hearn (wohearn@dpz.eu).

### Materials availability

This study did not generate new unique materials.

### Data and code availability

• Data have been deposited in an OSF archive and are publicly available as of the date of publication. The link is listed in the key resources table.
• All original code has been deposited in an OSF archive and is publicly available as of the date of publication. The link is listed in the key resources table.
• Any additional information required to reanalyze the data reported in this article is available from the lead contact upon request.

## Acknowledgments

We want to thank the Direction des Parcs Nationaux (DNP) and the Ministère de l’Environnement et de la Protection de la Nature (MEPN) du Sénégal for approval to conduct this study in the Parc National du Niokolo-Koba (PNNK), as well as all present and past Conservateurs of the park. We are grateful to the CRP team members of the last nine years for their help with the data collection. This research was funded by the 10.13039/501100001659Deutsche Forschungsgemeinschaft – Project-ID 454648639 SFB 1528 , Cognition of Interaction“ and Project-ID 254142454/GRK 2070 “Understanding Social Relationships.” The University of Göttingen functioned as the paying institution via the DEAL agreement. Support by the Leibniz ScienceCampus “Primate Cognition” (Audacity Fund AF2020_03) is gratefully acknowledged.

## Author contributions

W.O., F.D.P., and J.F. developed the study’s concept and design; W.O. extracted data from the meat-eating event records; F.D.P. curated and extracted the demographic and behavioral data, and assisted with data preparation; W.O., F.D.P., C.N., and R.M. designed and conducted the analysis with supervision from J.F.; W.O. drafted the article; and W.O., F.D.P., C.N., R.M., and J.F. reviewed the article and provided final approval of the submitted version.

## Declaration of interests

The authors declare no competing interests.

## STAR★Methods

### Key resources table

**Table:** 

REAGENT or RESOURCE	SOURCE	IDENTIFIER
**Deposited data**		

Cleaned data and code	OSF Archive	OSF: https://osf.io/n64ep/
Video examples of meat eating	OSF Archive	OSF: https://osf.io/n64ep/

**Experimental models: Organisms/strains**		

Wild Guinea baboons: 465 individually identified males and females	Not Applicable	Not Applicable

**Software and algorithms**		

R v4.2.0	R Core Team 2024	https://cran.r-project.org/
Stan v2.35.0	Stan Development Team 2024	https://mc-stan.org/about/
R Package cmdstanr	Gabry et al. 2024	https://stan-dev.r-universe.dev/cmdstanr
R package clmm	Christensen 2019	https://cran.r-project.org/web/packages/ordinal/vignettes/clmm2_tutorial.pdf

### Experimental model and subject details

#### Subjects

Subjects were 61 male and 42 female wild Guinea baboons (*Papio papio*) living in Niokolo-Koba National Park, Senegal. All individuals were either adults or subadults and belonged to 13 different parties, eight of them core focal parties.

#### Field site

Field work for this study was based at the field station “Centre de Recherche de Primatologie (CRP) Simenti” (13°01’34” N, 13°17’41” W) in the Niokolo-Koba National Park, Senegal. The site lies next to the Gambia River, where multiple seasonal wetlands (Mare) occur in depressions alongside the river. The climate is highly seasonal, with a dry season from November until May and a rainy season from June until October.^19^ The home ranges of the parties covered, on average, 30.3 km^2^ of largely overlapping territories (estimated 95% kernel density).^24^ Between April 2014 and June 2023, we entered 465 individually identified animals into our database, including adults, subadults, juveniles, and infants of our core focal parties; some identified animals resided in adjacent parties. Baboons in the focal parties were fully habituated and individually identifiable by natural markings, body shape, size, and radio collars.

Guinea baboons live in a multilevel society, the base of which are stable ‘units’ comprised of a single reproductive male and one to several females with their young.^55^^,^^56^ Three to four units consistently and frequently associate with one another to form the ‘party’ level, and two to three parties regularly aggregate into ‘gangs’ with overlapping home ranges.^19^^,^^35^^,^^56^ At the party level, males form enduring social bonds with other males, with whom they often share a higher degree of relatedness.^17^

#### Ethics declaration

This research was conducted within the regulations set by Senegalese authorities and the guidelines for the ethical treatment of nonhuman animals set down by the Association for the Study of Animal Behaviour.^57^

### Method details

#### Observational data collection

Demographic, observational, and meat-eating data were recorded by researchers from April 2014 to June 2023. We performed behavioral observations of adults and subadults in eight focal parties by conducting 20-minute focal follows^58^ balanced between subjects and time of day for an average of six protocols per individual and month. During protocols, we continuously recorded focal animal activity (i.e., moving, feeding, resting, and socializing) and all occurrences of social behaviors such as approaching within one meter, grooming, contact-sit, and greeting.^59^ Observers recorded all behavioral observations on electronic forms created using the Pendragon software (Pendragon Software Corporation, USA) running on cellular phones (Samsung Note 2 and Gigaset GX290).

During each observation day, we recorded census information about demographic changes (i.e., births, deaths, dispersal, and presence/absence), health status, female reproductive state, and the identity of other parties nearby.^59^ We monitored female-male associations and unit composition daily by collecting data on female-male interactions (i.e., frequency of copulations, grooming bouts, contact-sit bouts, greetings, aggression events, and duration of grooming and contact-sit bouts). We used female-male interaction rates to verify daily unit composition within each study party,^17^^,^^60^ based on previous findings that female Guinea baboons interact with their primary male at significantly higher rates and mate almost exclusively with him.^56^

#### Meat eating event data collection

Prey capture and meat-eating events were observed opportunistically and, when possible, were recorded with video cameras (handheld Panasonic HC-X909 and GoPro Hero8; examples of meat eating videos are available in an OSF archive listed in the key resources table). At the same time, the observer verbally described the unfolding scenario and recorded the audience composition. Individuals who caught the prey and were first to possess the carcass were identified and recorded. Individuals who possessed a carcass or from whom meat was transferred were labeled ‘possessors,’ while the individuals receiving meat were labeled ‘recipients.’ Only adults and subadults were recorded as possessors or recipients of meat. Individuals who did not catch the prey themselves could acquire meat through four transfer types: ‘passive sharing,’ ‘scavenging,’ ‘succeeding,’ or ‘stealing,’ in line with previous descriptions of meat eating in Guinea baboons.^16^ Passive meat sharing^38^ was characterized as the tolerated transfer of a defensible food item.^61^ We described all transfers as ‘passive meat sharing’ when a recipient took meat from the carcass itself or the ground just around the carcass (< 2 m) while the owner was still feeding and in the absence of aggression and submission by either individual. ‘Scavenging’ was scored when recipients acquired pieces of meat left behind (>2 m) when the possessor moved the carcass. ‘Succeeding’ occurred when a recipient moved into the feeding position recently vacated by the owner (formerly ‘supplant’ see ref. ^16^). ‘Stealing’ was noted when the recipient forcefully took possession of the carcass despite resistance by the owner. Transfers were labeled ‘unknown’ when meat passed from the possessor to the recipient, but the transfer type could not be observed (e.g., the subject was filmed from the back).

### Quantification and statistical analysis

#### Model 1: Transfer tolerance and societal level

In the first model, we sought to measure how meat moved through the multi-level society of Guinea baboons. Our first question was: “How is the degree of tolerance – reflected by different meat transfer types – related to the level of the society at which the transfer occurs and the sex of the possessor or the recipient?”. To answer this question, we fitted an ordinal (i.e., cumulative logit-link) mixed model^36^ using the function clmm of the R package ordinal version 2023.12-4.^62^ This model allowed us to include meat transfer type as an ordinal response variable measuring three different types of transfers from most to least tolerant (share > scavenge/succeed > steal). We combined ‘scavenge’ and ‘succeed’ into one category because both are neutral transfer types that occur without direct interaction with the meat possessor. In contrast, ‘stealing’ is a transfer type that occurs in the face of opposition from the possessor, while ‘passive sharing’ only happens with the possessor’s tolerance.

The primary predictor of the model was ‘social level’, a categorical variable with three levels (same unit/same party, different unit/same party, different unit/different party), which described three types of social-level-based relationships individuals could share within the Guinea baboon society (i.e., unit, party, gang). We also included the sex of the possessor, the sex of the recipient, and their interaction as categorical predictors with the expectation that males transferring tolerantly with females would be most frequent. Finally, we included Possessor-, Recipient-, Dyad-, and Event-IDs as random intercept effects to account for repeated observations. Random slopes were not included because they were not theoretically identifiable for any of the predictor-grouping factor pairs.^63^^,^^64^ We also fitted a reduced version of the model (see Supplements) without the interaction between the possessor and recipient sex to draw inferences about their effects on their own.^65^ Ordinal models assume “proportional odds”, meaning that they consider the spacing between adjacent categories, i.e., share to scavenge/succeed, and scavenge/succeed to steal, are equal in log-odds space. However, to our knowledge, for ordinal mixed models with multiple grouping factors, it is not straightforward to assess whether the model’s assumptions are fulfilled. To address this question of spacing between adjacent categories, we fitted two logistic models using the data of the ordinal model, but with the response dichotomized once between share/scavenge/supplant (0) or steal (1) and once between share (0) or scavenge/supplant/steal (1). The fixed and random effects structure of these models was identical to that of the ordinal model. We then compared the fixed effects estimates of these logistic models with those of the ordinal model. We found them to be roughly similar, suggesting that the proportional odds assumption was not strongly violated (Table S4).

After fitting the ordinal model, as an overall test of our main predictors and to avoid “cryptic multiple testing”, we conducted full-null model comparisons,^64^ whereby the null model lacked the primary predictors (i.e., social level, owner sex, and recipient sex) but was otherwise identical to the full model. The comparison used a likelihood ratio test.^66^ We assessed model stability on the level of the estimated coefficients by excluding individual levels of the random effects one at a time^67^ using a function written by RM. The model was of acceptable stability. We determined 95% confidence intervals of model estimates and fitted values using a parametric bootstrap (N=1.000 bootstraps) using the function written by RM. We fitted the models in R (version 4.2.0^68^).

#### Model 2: Transfer probability and relationship strength

Our second question was: “Are close social associates of meat possessors more likely to receive meat transfers?”. To answer this question, we built a custom Bayesian model fitted using Stan (v. 2.35.0^69^) and its R interface cmdstanr (v. 0.8.1.9000^70^). The model consists of two major sections. In the first, we estimate the sociality components from the behavioral data. The results of this step are the individual gregariousness and dyadic affinity^37^ values that are needed in the subsequent steps. These values are the primary predictor values for the subsequent model(s). The second section deals with modeling probabilities of meat transfers and consists of three independent sub-models. The most important of these sub-models investigates which individual gets the meat next as a function of the dyadic affinity between the possessor and the audience members. The two other sub-models represent internal ‘controls’, or sanity checks. Here, we modeled audience size as a function of individual gregariousness of the possessor and audience composition as a function of affinity between possessor and party members.

##### Sociality axes: Gregariousness and affinity

In the first step, we estimated two sociality components: individual ‘gregariousness,’ defined as an individual’s general tendency to interact with any group member regardless of their identity, and dyadic ‘affinity,’ defined as the preference of two individuals to interact specifically with one another. We used data from three behaviors (approaches, grooming, and contact sitting) to estimate the two sociality dimensions separately for the 90 days preceding each meat-eating event. To avoid redundancies with the other behaviors, approaches were only included if no other social behaviors (positive or negative) took place in the following ten seconds. Approaches were modeled as Poisson distributed with an offset for observation effort. Grooming and contact sitting were modeled as continuous proportions (‘proportion time spent grooming/in contact’) following a Beta distribution. We added a tiny amount of uniform noise to the grooming and contact sitting data to ensure that all values were larger than 0. The following equation describes the model for one event:

(Equation 1) $$\begin{array}{rr} {\text{approach}}_{ij} & ∼\text{Poisson}(\exp\left(\mu_{ij}+b_{0_{\text{approach}\;}}+\log\left({\;\text{obseff}\;}_{ij}\right)\right)\\ {\text{groom}}_{ij} & ∼\text{Beta}\left({\text{logit}}^{-1}\left(\mu_{ij}+b_{0_{\text{groom}\;}}\right),\phi_{\text{groom}\;}\right)\\ {\text{contact}}_{ij} & ∼\text{Beta}\left({\text{logit}}^{-1}\left(\mu_{ij}+b_{0_{\text{contact}\;}}\right),\phi_{\text{contact}\;}\right)\\ \mu_{ij} & =\sqrt{0.5}*\left(g_{i}+g_{j}\right)+a_{ij}\\ g & ∼\text{Normal}\left(0,\sigma_{g}^{2}\right)\\ a & ∼\text{Normal}\left(0,\sigma_{r}^{2}\right) \end{array}$$

We set the following priors:

(Equation 2) $$\begin{array}{rr} \sigma_{g}^{2} & ∼\text{Exponential}\left(1\right)\\ \sigma_{a}^{2} & ∼\text{Exponential}\left(1\right)\\ b_{0_{\text{approach}\;}} & ∼\text{Normal}\left(0,1\right)\\ b_{0_{\text{groom}\;}} & ∼\text{Normal}\left(-6,2\right)\\ b_{0_{\text{contact}\;}} & ∼\text{Normal}\left(-6,2\right)\\ \phi_{\text{groom}\;} & ∼\text{Exponential}\left(0.1\right)\\ \phi_{\text{contact}\;} & ∼\text{Exponential}\left(0.1\right) \end{array}$$

From this model we obtained estimates (posterior distributions) for individual gregariousness and dyadic affinities that could be mapped onto features of meat transfers. In other words, we used the full posteriors of these parameters as predictors in the subsequent models.

##### Meat eating events

The second step consisted of three sub-models: one primary model and two control models. Each sub-model concerned a different feature of meat transfers (i.e., audience size, audience composition, dyadic affinity of audience members), with either gregariousness or affinity as the primary predictor. We used the full posterior distributions of either gregariousness or affinity for all sub-models as a predictor, i.e., we propagated the uncertainty in estimating these sociality features downstream into the models of meat transfer. The final sample for the sub-models included 232 meat transfers. We removed 48 steal transfers from the initial sample of 314 because we expected that dyadic affinity would not affect intolerant transfer types. We removed 34 other transfers for which we could not calculate affinity: in 15 cases, they occurred between cross-party dyads, and in 19 cases, we lacked focal observations in the party for 90 days before the meat-eating event.

###### Audience size

We began with modeling the audience size, i.e. the number of individuals that were present during a meat transfer. We modeled this as a truncated Poisson variable because there was always at least one individual present. The key predictor was the individual gregariousness value g_*indi*_ of the possessor on the date corresponding to the meat-eating event (as estimated in step 1). We estimated an intercept (c_0_) and a slope for the effect of gregariousness (c_1_). We also estimated varying intercepts for possessor and party identities.

(Equation 3) $$\begin{array}{rr} {\;\text{audience}\;\text{size}\;}_{i} & ∼{\text{Poisson}}^{+}\left(\exp\left(\mu_{i}\right)\right)\\ \mu_{i} & =c_{0}+c_{0_{\text{possessor}\;}}+c_{0_{\text{party}\;}}+c_{1}\times g_{{ind}_{i}}\\ c_{0_{\text{party}\;}} & ∼\text{Normal}\left(0,\sigma_{\text{party}\;}^{2}\right)\\ c_{0_{\text{possessor}\;}} & ∼\text{Normal}\left(0,\sigma_{\text{possessor}\;}^{2}\right) \end{array}$$

We set the following priors:

(Equation 4) $$\begin{array}{rr} \sigma_{\text{party}\;}^{2} & ∼\text{Exponential}\left(1\right)\\ \sigma_{\text{possessor}\;}^{2} & ∼\text{Exponential}\left(1\right)\\ c_{0} & ∼\text{Normal}\left(0,1\right)\\ c_{1} & ∼\text{Normal}\left(0,1\right) \end{array}$$

###### Audience composition

For each meat transfer, we modeled the audience composition as a multinomial distribution. Audience composition was defined as the identities of the individuals present at a transfer out of all individuals that could have been present, i.e. the adult party members on the date of the meat-eating event. For modeling, we considered a vector of 0 and 1 to represent potential audience and actual audience, respectively. Importantly, the length of this vector could vary from transfer to transfer. The predictor was the dyadic affinity of all potential audience members with the possessor (within the party). This predictor was multiplied with our parameter of interest (c_affi_, see Figure S1). The following equation describes the model for one meat transfer *i*. a_*ij*_ is the dyadic affinity between possessor and all potential audience members.

(Equation 5) $$\begin{array}{cc} {\;\text{present}\;\text{in}\;\text{audience}\;}_{i} & ∼\text{Multinomial}\;\left(\text{softmax}\left(\mu_{i}\right)\right)\\ \mu_{i} & =c_{\text{affi}\;}\times a_{ij} \end{array}$$

We set the following priors:

(Equation 6) $$c_{\text{affi}\;}∼\text{Normal}\left(0,1\right)$$

###### Meat transfer

The primary sub-model quantified the relationship between the dyadic affinity of all audience members with the meat possessor and the probability that one of the audience members would receive meat from the possessor. We pruned the data for this model, limiting it to events with at least two individuals in the audience, resulting in 148 transfers. As before, the response came from a multinomial distribution and was a vector of one 1 and a varying number of 0s given audience size for each transfer. We derived the probability vector for the multinomial likelihood from a linear predictor, i.e., the dyadic affinity values of all audience members with the possessor. This linear predictor was then multiplied by the slope parameter of interest (b_affi_) per transfer. If that slope were positive, it would indicate that higher affinities were associated with higher probabilities of obtaining meat (see Figure S1). The length of the linear predictor varied from event to event to reflect the different audience sizes. For instance, if we had an event with an audience of three, the linear predictor would consist of three affinities and the response of a vector of one 1 and two 0 (one individual obtained the meat, two did not; see Figure 3). In this model, we also included a parameter for partner sex (b_*sex*_) and a varying intercept for the partner’s ID. This last term reflects the possibility that individuals differed in their propensity to receive meat irrespective of their dyadic affinity with the meat possessor.

(Equation 7) $$\begin{array}{cc} {\text{transfer}\;\left(\text{obtained}\;\text{meat}\right)}_{i} & ∼\text{Multinomial}\left(\text{softmax}\left(\mu_{i}\right)\right)\\ \mu_{i} & =b_{\text{affi}\;}\times a_{ij}+b_{\text{sex}\;}\times \text{sex}+b_{0\;\text{partner}\;}\\ b_{0\;\text{partner}\;} & ∼\text{Normal}\left(0,\sigma_{\text{partner}\;}^{2}\right) \end{array}$$

We set the following priors:

(Equation 8) $$\begin{array}{rr} b_{\text{affi}\;} & ∼\text{Normal}\left(0,1\right)\\ b_{\text{sex}\;} & ∼\text{Normal}\left(0,1\right)\\ \sigma_{\text{partner}\;}^{2} & ∼\text{Exponential}\left(1\right) \end{array}$$

#### Modeling approach

The main reasons for fitting model 1 in a frequentist and model 2 in Bayesian framework was ease of implementation and control for multiple testing. In model 1 we had three main effects (social level, sex of the possessor, the sex of the recipient, and the interaction between recipient and possessor sex). Such a model poses a 'cryptic' multiple testing problem^64^ which can be easiest addressed by a full-null model comparison which is only readily available in a frequentist framework. Model 2, in turn, comprised only a single predictor of interest (social association), meaning there was no issue with multiple testing. At the same time, we wanted to propagate the uncertainty from estimating dyadic affinity into downstream estimates of its effect on the probability of meat transfers, which was by far easiest to implement in a Bayesian framework. Hence, we implemented the two models in the two different frameworks.
