# Supplementary material for: Meat transfer patterns reflect the multi-level social system of Guinea baboons
Source: iScience. 2025 Sep 20;28(11):113619. doi: 10.1016/j.isci.2025.113619 (PMC12547275; doi:10.1016/j.isci.2025.113619)
Supplement: Document S1. Figures S1, S2, and Tables S1–S5 [file mmc1.pdf]

iScience, Volume *28*

Supplemental information

Meat transfer patterns reflect

the multi-level social system of Guinea baboons

William J. O'Hearn, Christof Neumann, Roger Mundry, Federica Dal Pesco, and Julia Fischer

Supplemental Methods

Meat transfer patterns reflect the multi-level social system of Guinea baboons

This file includes:
Tables S1 to S5
Figure S1 to S2

Table S1: Prey animals were captured and consumed by Guinea baboons in the 109 meat-eating events recorded by CRP Simenti within Niokolo-Koba National Park, Senegal, between April 2014 and June 2023.

Prey type	Number of events
Bushbuck (*Tragelaphus scriptus*)	65
Unidentified bird	13
Red-flanked duiker (*Cephalophus rufilatus*)	5
African savanna hare (*Lepus victoriae*)	5
Mouse	5
Helmeted guineafowl (*Numida meleagris*)	3
Oribi (*Ourebia ourebi*)	2
Ahanta francolin (*Pternistis ahantensis*)	2
Kob (*Kobus kob*)	1
African striped ground squirrel (*Euxerus erythropus*)	1
Stone Partridge (*Ptilopachus petrosus*)	1
Western tinkerbird (*Pogoniulus coryphaea*)	1
Grey-headed bristlebill (*Bleda canicapillus*)	1
Unidentified	4

Table S2: Ordinal Model Output (Full Model).

Term	$\beta^{(1)}$	SE	Lower CI	Upper CI	χ^2	df	P	Min	Max
steal|scavenge-succeed	−0.70	0.71	−2.11	0.58				−0.98	−0.33
scavenge-succeed|passive-sharing	2.98	0.70	1.74	4.67				2.79	3.29
possessor male	0.36	0.48	−0.58	1.38				0.20	0.52
recipient male	−1.02	0.63	−2.31	0.23				−1.25	−0.65
possessor male:recipient male	0.74	0.66	−0.64	2.09	1.19	1	0.260	0.45	1.04
social level different unit/same party	1.32	0.46	0.47	2.36	20.27	2	$< 0.001^{(2)}$	1.06	1.55
social level same unit/same party	2.44	0.55	1.50	3.74	20.27	2		2.25	2.71

(1) Indicated are estimates, together with their standard errors, 95% confidence limits, significance tests, and the range of estimates obtained when dropping the individual levels of random effects factors from the data, one at a time.
(2) This represents the overall effect of social level on transfer type.

Table S3: Ordinal Model Output (Reduced Model Lacking the Non-Significant Interaction Between Actor Sex and Recipient Sex).

Term	$\beta^{(1)}$	SE	Lower CI	Upper CI	χ^2	df	P	Min	Max
steal|scavenge-succeed	−0.52	0.68	−1.85	0.74				−0.84	−0.11
scavenge-succeed|passive-sharing	3.16	0.66	2.04	4.85				2.97	3.52
possessor male	0.71	0.37	−0.02	1.60	3.62	1	0.057	0.58	0.91
recipient male	−0.44	0.38	−1.19	0.29	1.35	1	0.246	−0.67	−0.25
social level different unit/same party	1.22	0.44	0.42	2.24	19.25	2	$< 0.001^{(2)}$	0.99	1.45
social level same unit/same party	2.35	0.54	1.38	3.66	19.25	2		2.17	2.60

(1) Indicated are estimates, together with their standard errors, 95% confidence limits, significance tests, and the range of estimates obtained when dropping the individual levels of random effects factors from the data, one at a time.
(2) This represents the overall effect of social level on transfer type.

Table S4: Comparison of model estimates to check assumption of proportion odds.

Term	Ordinal	Logistic $1^{(1)}$	Logistic $2^{(2)}$
social level: same unit/same party	2.45	1.97	3.34
social level: different unit/same party	1.32	1.50	1.39
owner sex: M	0.36	−0.66	1.03
recipient sex: M	−1.02	−2.27	−1.24
owner sex M:recipient sex M	0.74	1.40	2.05

(1) The response of logistic model 1 is scavenge/succeed (1) or steal (0).
(2) The response of logistic model 2 is share (1) or scavenge/supplant/steal (0).

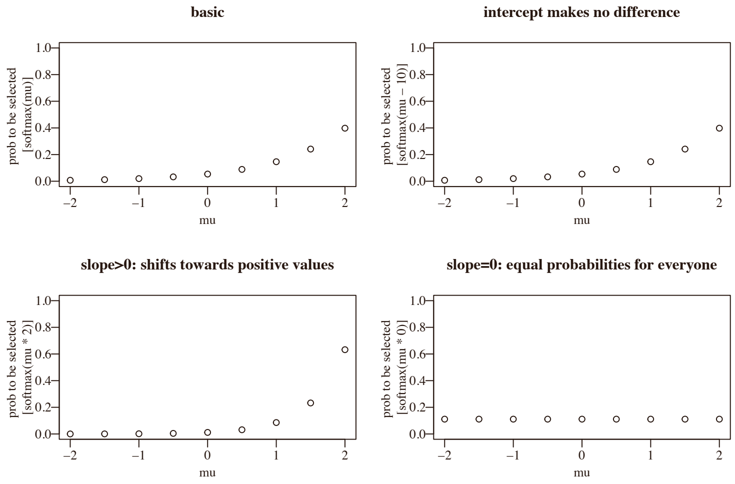

Figure S1: Illustrating modeling probability vectors with softmax. The softmax function is defined as $\text{softmax}(x) = \frac{e^x}{\sum e^x}$. In a multinomial model we deal with probabilities that sum to 1 across all possibilities in a given meat transfer. We parameterized our meat transfer model such that these probabilities come from a linear (and unconstrained) predictor (LP), which is easier to handle internally. Linking the unconstrained LP to probabilities is done by the softmax function (top left). In terms of modelling, we work with the LP. For example, adding an intercept will not change the probabilities (top right). Importantly, we add a slope parameter to our model, i.e., we multiply the LP by a scalar (this is our slope parameter). If that slope were zero, we obtain equal probabilities, meaning the LP does not covary with the probabilities (bottom right). If the slope were positive, we would see that positive LP values get larger probabilities and negative LP values approach zero probabilities (bottom left).

Table S5: Numeric results of key model parameters.

variable	mean	median	q5	q95	rhat	ess_bulk	ess_tail
audience size (intercept)	0.36	0.37	0.05	0.65	1.00	3181	2909
audience size (greg slope)	0.13	0.13	-0.30	0.57	1.00	2990	3254
composition (affinity)	0.14	0.14	0.03	0.24	1.00	6396	3424
transfer (affinity)	0.30	0.30	0.04	0.57	1.00	5351	3599
transfer (sex)	0.12	0.10	-0.35	0.61	1.00	4998	2998

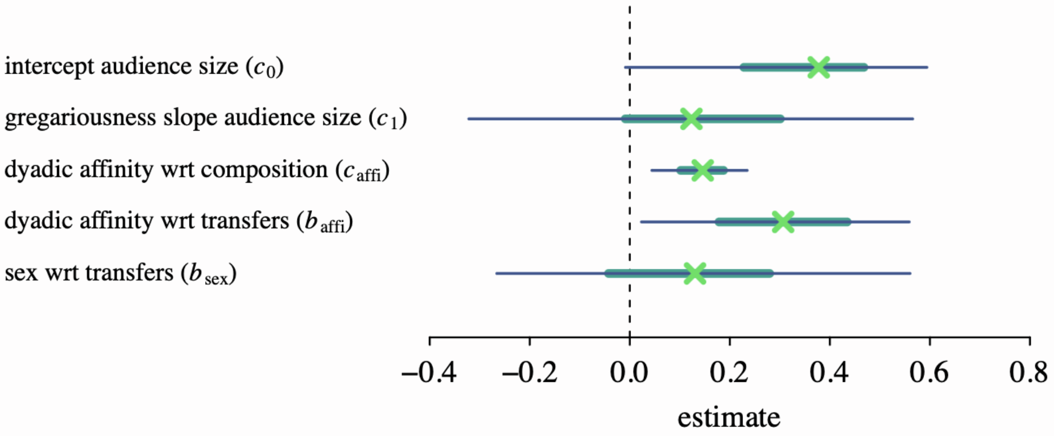

Figure S2: Visual summaries of key posterior distributions.
